# Secretory leukocyte protease inhibitor influences periarticular joint inflammation in *B. burgdorferi* -infected mice

**DOI:** 10.1101/2024.11.24.625079

**Published:** 2024-11-25

**Authors:** Qian Yu, Xiaotian Tang, Thomas Hart, Robert Homer, Alexia A. Belperron, Linda K. Bockenstedt, Aaron Ring, Akira Nakamura, Erol Fikrig

## Abstract

Lyme disease, caused by *Borrelia burgdorferi* , is the most common tick-borne infection in the United States. Arthritis is a major clinical manifestation of infection, and synovial tissue damage has been attributed to the excessive pro-inflammatory responses. The secretory leukocyte protease inhibitor (SLPI) promotes tissue repair and exerts anti-inflammatory effects. The role of SLPI in the development of Lyme arthritis in C57BL/6 mice, which can be infected with *B. burgdorferi* , but only develop mild joint inflammation, was therefore examined. *SLPI* -deficient C57BL/6 mice challenged with *B. burgdorferi* had a higher infection load in the tibiotarsal joints and marked periarticular swelling, compared to infected wild type control mice. The ankle joint tissues of *B. burgdorferi-* infected *SLPI* -deficient mice contained significantly higher percentages of infiltrating neutrophils and macrophages. *B. burgdorferi* -infected *SLPI* -deficient mice also exhibited elevated serum levels of IL-6, neutrophil elastase, and MMP-8. Moreover, using a recently developed BASEHIT ( **BA** cterial **S** election to **E** lucidate **H** ost-microbe **I** nteractions in high **T** hroughput) library, we found that SLPI directly interacts with *B. burgdorferi* . These data demonstrate the importance of SLPI in suppressing periarticular joint inflammation in Lyme disease.

## Full Text Availability

The license terms selected by the author(s) for this preprint version do not permit archiving in PMC. The full text is available from the preprint server.

